# Comparative Transcriptome Analysis Reveals Sexually Dimorphic Gene Expression in the Gonads of *Brachymystax tsinlingensis* Li

**DOI:** 10.3390/ani13233690

**Published:** 2023-11-29

**Authors:** Ling Huang, Huan Ye, Huamei Yue, Xiaoqian Leng, Rui Ruan, Hao Du, Chuangju Li, Jinming Wu

**Affiliations:** Key Laboratory of Freshwater Biodiversity Conservation, Ministry of Agriculture and Rural Affairs, Yangtze River Fisheries Research Institute, Chinese Academy of Fishery Sciences, Wuhan 430223, China; huangling@yfi.ac.cn (L.H.); yehuan@yfi.ac.cn (H.Y.); yhuam@yfi.ac.cn (H.Y.); lengxiaoqian@yfi.ac.cn (X.L.); ruanrui@yfi.ac.cn (R.R.); duhao@yfi.ac.cn (H.D.)

**Keywords:** *Brachymystax tsinlingensis* Li, testis and ovary, transcriptome analysis, sex-biased genes

## Abstract

**Simple Summary:**

*Brachymystax tsinlingensis* Li has become an endangered species due to human activities and habitat degradation. However, little is known about the genes regulating its gonadal development. This study presents a high-quality gonadal transcriptome of *B. tsinlingensis* Li. A total of 22,864 differentially expressed genes between male and female were identified. Numerous sex-related differentially expressed genes, such as *ccnb1*, *zp3*, *bmp15*, *dmrt1*, and *psmc3ip,* were identified. Some signaling pathways involved in gonadal development, such as genes involved in base excision repair, the notch signaling pathway, neuroactive ligand-receptor interaction, the VEGF signaling pathway, and the estrogen signaling pathway, were found to be enriched through analysis using the Kyoto Encyclopedia of Genes and Genomes. Our results provide molecular data to support a better understanding of sexual development in *B. tsinlingensis* Li.

**Abstract:**

*Brachymystax tsinlingensis* Li is an endangered cold-water salmonid fish native to China. This study aimed to identify sex-related genes and biological pathways via gonadal transcriptome sequencing of *B. tsinlingensis* Li. A total of 167,904 unigenes were identified with an average length of 836 bp and an N50 of 1452 bp, of which 84,977 (50.61%) unigenes were successfully annotated in six major databases. Comparative transcriptome analysis identified 22,864 differentially expressed genes (DEGs), of which 17,231 were up-regulated (male-biased genes, mDEGs) and 5633 were down-regulated (female-biased genes, fDEGs). Several DEGs associated with gonadal development were found through Gene Ontology enrichment analysis, such as *ccnb1*, *zp3*, *bmp15*, *dmrt1*, and *psmc3ip.* Signaling pathways related to gonadal development were found to be enriched through analysis using the Kyoto Encyclopedia of Genes and Genomes Pathway database, such as genes involves in base excision repair, the notch signaling pathway, neuroactive ligand-receptor interaction, the VEGF signaling pathway, and the estrogen signaling pathway. In addition, mRNA expression levels of 19 DEGs were determined to validate the reliability of the transcriptomic data by quantitative real-time polymerase chain reaction. These results revealed genes and signaling pathways potentially involved in gonadal development in *B. tsinlingensis* Li and provided basic molecular data for future research on reproductive regulation and breeding of *B. tsinlingensis* Li.

## 1. Introduction

*Brachymystax tsinlingensis* Li, which belongs to the Salmonidae family, is widely distributed in southern China. It originated from Siberia during the fourth glacial period and migrated to the cold-water streams of the Hei River, Shitou River, Xushui River, and Taibai River in the Tsinling Mountains as the glacier advanced [1]. However, due to natural and human factors, such as global warming, environmental destruction, and overfishing, the wild population of this species has declined severely and only 10% of its historical population currently remains. This species was listed as a second-class nationally protected animal in China in 1988 [2,3]. Artificial propagation and release is one effective method by which to restore the wild population of *B. tsinlingensis* Li. However, large-scale artificial propagation of this species has not been successful, partly because of our limited knowledge of the molecular mechanisms governing reproduction in *B. tsinlingensis* Li.

Previous studies in *B. tsinlingensis* Li mainly focused on behaviours [4], molecular genetic markers [5], feed additives [6], and morphology and the ultrastructure of the sperm [7]. However, research on reproductive development in *B. tsinlingensis* Li is still lacking, although such research is essential for artificial propagation and further species conservation. Transcriptome sequencing technology has been widely used to study gene-expression profiles and molecular mechanisms with high efficiency and low-cost [8]. Comparative gonadal transcriptome analysis has been used to reveal molecular pathways and genes involved in gonadal development and reproduction in aquatic animals. In Hong Kong catfish (*Clarias fuscus*), many differentially expressed genes (DEGs) were found to be enriched using sex-related gene ontology (GO) terms and analysis through Kyoto Encyclopedia of Genes and Genomes (KEGG) pathways, such as oocyte maturation, androgen secretion, gonadal development and steroid biosynthesis [9]. The *dmrt1* gene has been revealed to play a dual role during testicular development in largemouth bass (*Micropterus salmoides*) [10]. In sea urchin (*Strongylocentrotus intermedius*), five DEGs related to gonadal quality have been identified [11]. Thus, the exploration of sex-related genes and biological pathways is an effective strategy by which to reveal sexual regulatory mechanisms.

This study presents a high-quality gonadal transcriptome of *B. tsinlingensis* Li. The gene-expression profiles of the testis and ovary were obtained, and candidate genes and biological pathways potentially related to gonadal development were further identified. Our study provides valuable insights for further research on the reproductive regulation and breeding of *B. tsinlingensis* Li.

## 2. Materials and Methods

### 2.1. Total RNA Extraction, cDNA Library Construction and Illumine Sequencing

A total of ten artificially cultured juvenile *B. tsinlingensis* Li (5 males and 5 females) collected from the Zhangjiachuan Malu *Brachymystax tsinlingensis* Li Artificial Breeding Farm in Gansu Province, China, were used for gonadal transcriptome analysis. The average length and weight of the male fish were 149.4 ± 11.52 mm and 58.4 ± 9.99 g, respectively, while those of the female fish were 143.2 ± 12.24 mm and 43.5 ± 11.03 g. After anesthesia, the gonads were sampled and stored in RNA Later (Sartorius, Gttingen, Germany) for RNA extraction; meanwhile, partial gonads were fixed in Bouin’s fixative solution (Scientific Phygene, Fuzhou, China), then subjected to gradient dehydration with ethanol, transparent tissue with xylene, paraffin dip, sectioning, and hematoxylin and eosin (HE) staining for tissue-structure evaluation. An RNeasy Plus Mini Kit (Qiagen, Dusseldorf, Germany) was used for extraction of total RNA. The total RNA was finally obtained by homogenizing gonadal tissue, removing the gDNA and purifying the RNA. The RNA quality was checked on a Nanodrop 2000 (Thermo Scientific, Waltham, MA, USA) and electrophoresis. High-quality RNA samples were sent to Meiji Biopharmaceutical Co., Ltd. (Shanghai, China) for construction of cDNA libraries and sequenced on the Illumina NovaSeq 6000 sequencing platform. All experimental procedures were carried out in accordance with the guiding principles for the care and use of laboratory animals set by the Yangtze River Fisheries Research Institute, Chinese Academy of Fishery Sciences, China.

### 2.2. De Novo Assembly and Functional Annotation

Fastp (version 0.19.5) was used to perform quality control for the original data. The numbers of raw reads, clean reads, raw bases and clean bases, as well as the error rate, Q30, and GC content were calculated to visualize the quality of library construction and sequencing. Generally, the error rate was below 0.1% and Q30 was above 80%. The clean reads have been submitted to the NCBI SRA database under the accession numbers SRR25744158, SRR25744157, SRR25744156, SRR25744155, SRR25744154, SRR25744153, SRR25744152, SRR25744151, SRR25744150, and SRR25744149.

The clean reads were then assembled into transcripts using Trinity (version v2.8.5), and the longest transcript for each gene was selected as the unigene. All unigenes were homology-annotated to the NCBI non-redundant protein database (NR), Swiss-Prot database, protein families database (Pfam), Evolutionary Genealogy of Genes: Non-supervised Orthologous Groups (eggNOG), GO, and KEGG, and the results were counted to obtain comprehensive functional information for the unigenes.

### 2.3. Identification of DEGs and Functional Enrichment Analysis

Gene-expression levels from each sample were estimated by RSEM (Version 1.3.1). The TPM (transcripts per million) algorithms were used to normalize mRNA expression levels. Sample correlation analysis and sample principal component analysis (PCA) were performed to validate the reliability of data and the rationality of the experimental design.

Based on the results for normalized reads, DESeq2 (Version 1.24.0) software was used to perform differential expression analysis of the genes. The *p*-values were corrected using the Benjamini–Hochberg (BH) method and represented as p-adjust. Unigenes with *p*-adjust < 0.001 & |log_2_FC| ≥ 4, which means that unigenes showing at least sixteen-fold expression difference between male and female were considered as DEGs. Compared with ovaries, DEGs with log_2_FC ≥ 4 were up-regulated in testes (male-biased genes, mDEGs), and those with log_2_FC ≤ −4 were down-regulated in testes (female-biased genes, fDEGs). Functional classification of DEGs was further analyzed by GO and KEGG annotation.

### 2.4. Quantitative Real-Time Polymerase Chain Reaction (qPCR) Verification

The accuracy of the RNA-Seq data was validated by qPCR. Gonad RNAs from five males and females used for transcriptomic sequencing were selected for gene-expression profiling. A total of 1 μg RNA was reverse-transcribed with the PrimeScript RT Reagent Kit With gDNA Eraser (Takara, Kyoto, Japan), and cDNA was diluted five-fold and stored at −20 °C until use. Quantitative real-time PCR was performed using a QuantStudio6 Flex PCR System (Applied Biosystems, CA, America) with PowerUp^TM^ SYBR^TM^ Green Master Mix. The sequence and amplification efficiency of the primers are listed in Table S1. *β-actin* was used as an internal reference. All samples were analyzed in triplicate, and relative gene expression was calculated using the 2^−ΔΔCt^ method.

### 2.5. Statistics Analysis

Statistical analysis was performed using Student’s *t*-test, with final data expressed as mean ± standard error. Differences were considered as significant when * *p* < 0.05, ** *p* < 0.01, and *** *p* < 0.001. All statistical data were plotted using GraphPad Prism 8.0. 

## 3. Results

### 3.1. Sex Identification and Histological Characteristics of Juvenile B. tsinlingensis Li Gonads

**Figure 1:**
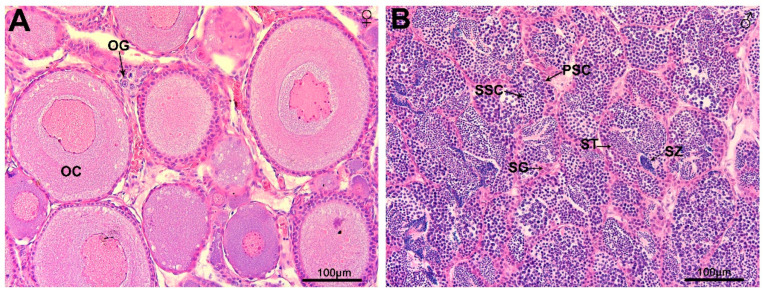
Histological sections of juvenile *Brachymystax tsinlingensis* Li gonads stained with hematoxylin and eosin. (**A**) Section of *B. tsinlingensis* Li ovary stained with HE; (**B**) Section of *B. tsinlingensis* Li testis stained with HE. OG, oogonia; OC, oocyte; SG, spermatogonia; PSC, primary spermatocytes; SSC, secondary spermatocytes; ST, spermatids; SZ, spermatozoa. Scale bar: 100 μm.

The histological characteristics of *B. tsinlingensis* Li gonads were determined by HE staining (Figure 1). The ovary had numerous primary oocytes and few oogonia, and the testes contained different types of germ cells, including spermatogonia, primary spermatocytes, secondary spermatocytes, spermatids, and spermatozoa.

### 3.2. Transcriptome Sequencing and Assembly

A total of 121.76 Gb of clean data was obtained. The Q30 values of the clean data were above 94.13%. The GC content was approximately 49.5%, with sequencing error rates no greater than 0.0251. A total of 167,904 unigenes were assembled. The minimum and maximum lengths of unigenes were 201 and 26,461 bp, respectively, with an N50 length of 1452 bp. The unigenes with sequence lengths exceeding 2000 bp and 3000 bp accounted for 10% and 5%, respectively. Moreover, the percentage of clean reads that could be mapped to the assembled transcripts ranged from 77.23% to 79.41%. Detailed information about transcriptome sequencing and assembly is shown in Table 1 and Table 2.

### 3.3. Functional Annotation

**Figure 2:**
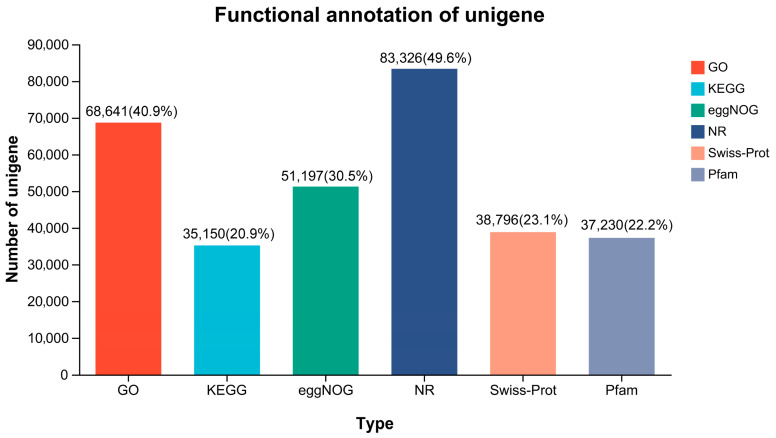
Numbers and percentages of annotated unigenes in different databases.

**Figure 3:**
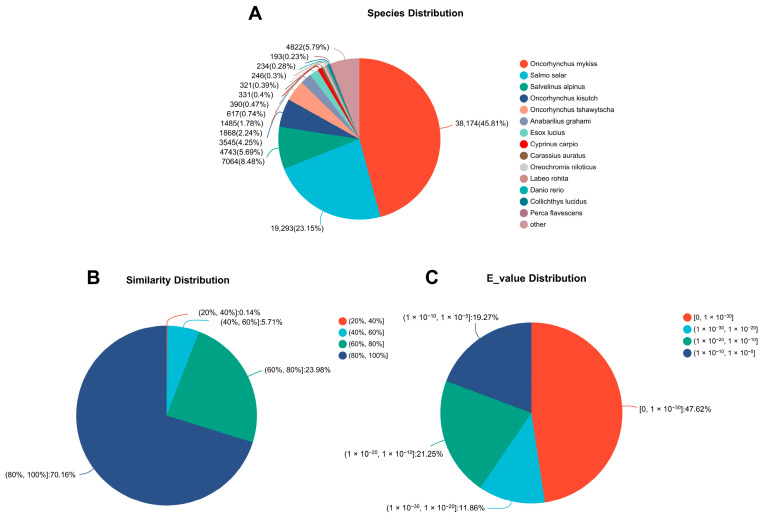
Summary of annotations from the NR protein database. (**A**) Species distribution; (**B**) Similarity distribution; (**C**) E-value distribution.

A total of 84,977 (50.61%) unigenes were annotated through six databases (NR, Swiss-Prot, Pfam, eggNOG, GO, and KEGG) (Figure 2). A large number of unigenes were matched to the NR and GO databases, accounting for 83,326 (49.6%) and 68,641 (40.9%), respectively. Based on the annotation in the NR database, 83,326 unigenes in *B. tsinlingensis* Li were matched to 165 species, of which 82,883 (99.5%) unigenes belonged to *Actinopteri* (Table S2). The greatest number of unigenes were homologous to genes of *Oncorhynchus mykiss* (38,174, 45.81%), followed by *Salmo salar* (19,293, 23.15%), *Salvelinus alpinus* (7064, 8.48%), etc. (Figure 3A). The number of unigenes with similarity above 80% was 58,464 (70.16%), and the number of unigenes with similarity of 60–80% was 19,981 (23.98%) (Figure 3B). Results with smaller E-values have higher confidence, and 47.62% of the E-values were distributed between 0 and 1 × 10^−30^ (Figure 3C), suggesting high accuracy and reliability of the RNA-Seq sequencing.

### 3.4. DEGs Identification and Enrichment Analysis

**Figure 4:**
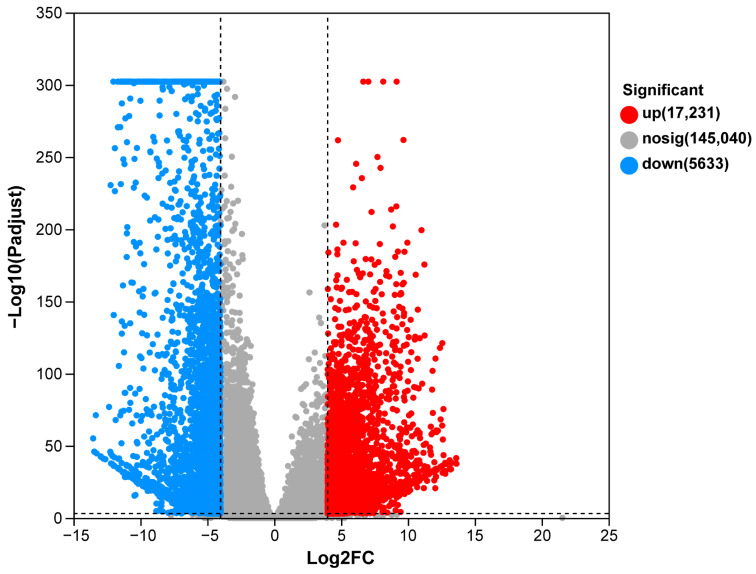
Volcano plot of DEGs in testis versus ovary.

To analyze the global similarities and differences between the testis and the ovary, correlation analysis and PCA were performed. The results showed that samples in the same group displayed similar expression patterns, while samples in different groups were clearly distinguishable (Figure S1), indicating the reliability and repeatability of the data. Comparative analysis were performed to identify sex-biased genes. A total of 22,864 DEGs, including 17,231 male-biased genes and 5633 female-biased genes, were identified (Figure 4). 

**Figure 5:**
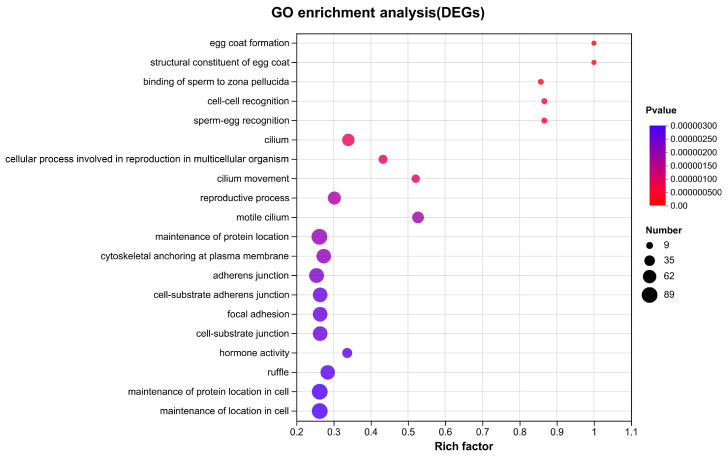
GO enrichment analysis of DEGs of *Brachymystax tsinlingensis* Li.

**Figure 6:**
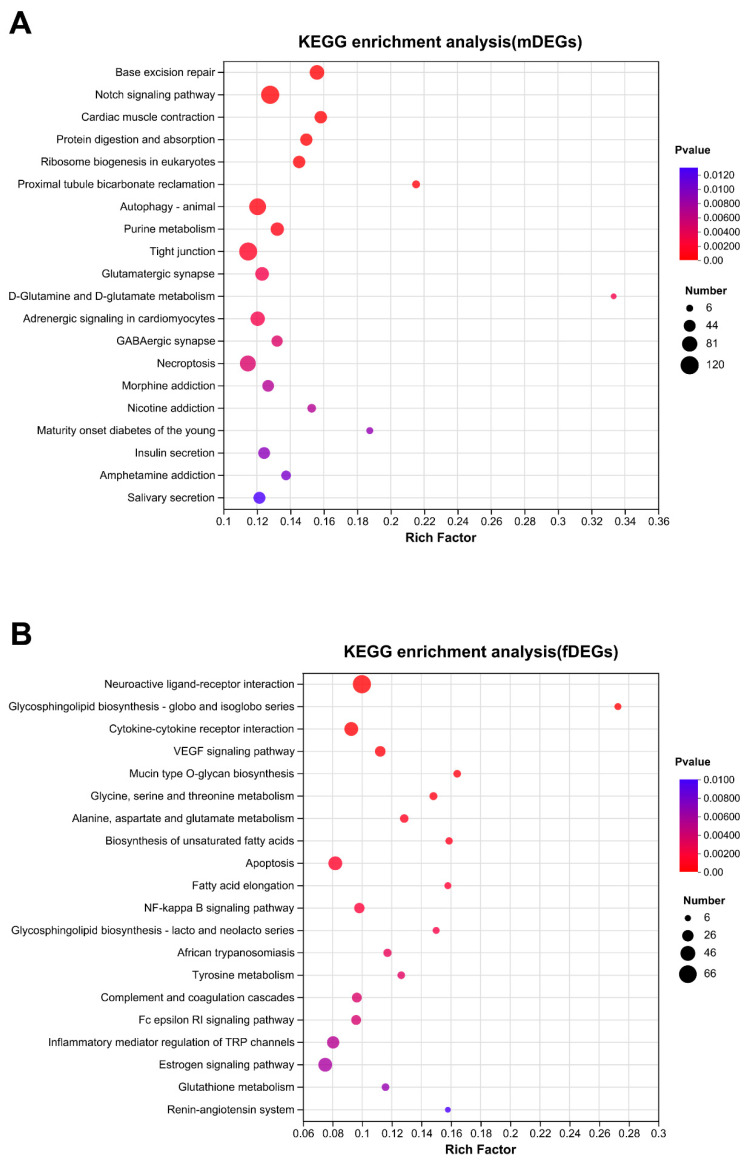
KEGG enrichment analysis of DEGs. (**A**) KEGG enrichment analysis of mDEGs; (**B**) KEGG enrichment analysis of fDEGs.

DEGs were further classified by GO and KEGG annotation to investigate their potential functions and metabolic pathways (Figure S2). The top 20 GO enrichment terms are shown in Figure 5. Sex-related terms, such as egg coat formation (BP, GO:0035803), structural constituent of egg coat (MF, GO:0035804), binding of sperm to zona pellucida (BP, GO:0007339), sperm-egg recognition (BP, GO:0035036), and reproductive process (BP, GO:0022414) were significantly enriched, with numerous reproductive-related genes included, such as *zp3*, *bmp15*, *ccnb1*, *psmc3ip*, *hsd17b3*, *piwi*, *sycp1*, *pvrl3*, *zent9*, *syce1*, *syce2*, *syce3*, *catsper2*, *cep4*, *igf1r*, *diaph2*, *plcz*, etc. A scatterplot of the top 20 enriched KEGG pathways of DEGs is presented in Figure 6. Several KEGG pathways related to reproduction were further identified, such as pathways related to base excision repair, the notch signaling pathway, cardiac muscle contraction, protein digestion and absorption, and ribosome biogenesis in eukaryotes in the testis (Figure 6A) and neuroactive ligand-receptor interaction, glycosphingolipid biosynthesis–globo and isoglobo series, cytokine-cytokine receptor interaction, the VEGF signaling pathway, and the estrogen signaling pathway in the ovary (Figure 6B). 

### 3.5. Validation of DEGs by qPCR

**Figure 7:**
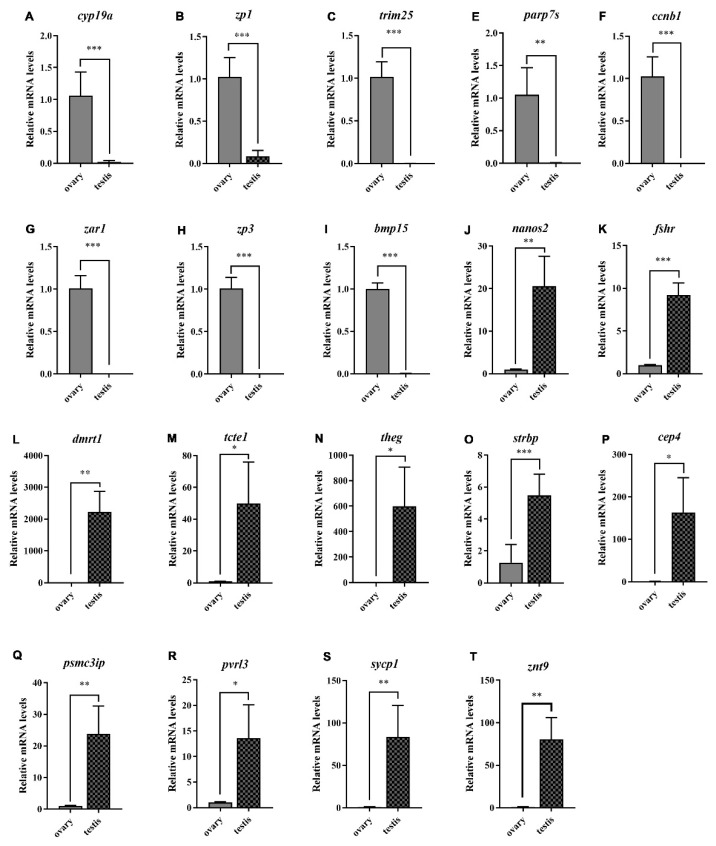
Quantitative real-time PCR validation of 19 DEGs between males and females. (**A**–**I**) qPCR validation of fDEGs; (**J**–**T**) qPCR validation of mDEGs. The data were expressed as the mean ± standard error of three independent experiments. * *p* < 0.05, ** *p* < 0.01, *** *p* < 0.001.

To validate the reliability of the RNA-seq results, the expression levels of 19 DEGs (8 fDEGs and 11 mDEGs) were determined by qPCR (Figure 7). The transcript levels of fDEGs, such as *cyp19a*, *zp1*, *trim25*, *parp7s*, *ccnb1*, *zar1*, *zp3*, and *bmp15*, were significantly higher than those in the testis, while the transcript levels of mDEGs, such as *nanos2*, *fshr*, *dmrt1*, *tcte1*, *theg*, *strbp*, *cep4*, *pamc3ip*, *pvrl3*, *sycp1*, and *znt9*, were significantly higher than those in the ovary. Overall, the qPCR results strongly confirmed the accuracy and reliability of transcriptome expression analysis conducted for *B. tsinlingensis* Li.

## 4. Discussion

The population of *B. tsinlingensis* Li has decreased significantly due to human activities and natural environmental changes, resulting in its being assigned endangered status. Artificial propagation and release is considered an important strategy through which to conserve and restore this species. However, little is known about the genes and regulatory mechanisms of reproductive development in *B. tsinlingensis* Li. This study was the first report of gonadal transcriptome analysis in *B. tsinlingensis* Li and revealed sex-related DEGs and regulatory pathways. To some extent, these results provide new insights into sexual development in *B. tsinlingensis* Li.

Various sex-biased genes, including both female-biased genes (*ccnb1*, *zp3*, *bmp15*) and male-biased genes (*psmc3ip*, *dmrt1*), were identified by analyzing the gene-expression profiles in the gonads of *B. tsinlingensis* Li. It has been reported that *ccnb1* has positive significance in the meiotic division of oocytes and in maintaining fertility [12,13]. *Zp3*, a critical component of the zona pellucida, plays a vital role in oocyte meiosis and maturation, and knockout of *zp3* leads to complete infertility [14]. Previous studies demonstrated that *bmp15* was necessary for the development and differentiation of oocytes [15,16]. In this study, *ccnb1*, *zp3,* and *bmp15* were highly expressed in the ovary (Figure 7F,H,I), implying that they have important functions during oocyte development and maintenance of the female phenotype in *B. tsinlingensis* Li.

Some testicular genes of *B tsinlingensis* Li were also found. The *dmrt1* gene exists on the Y chromosome in the males of some fishes [17,18] and regulates the differentiation and proliferation of male germ cells and germline stem cells [19,20,21,22]. Moreover, several studies have found that *dmrt1* inhibits the sexual switch from male to female [23,24,25]. The *psmc3ip* gene is essential for homologous recombination during meiotic division, and deficiency of *psmc3ip* results in azoospermia [26,27]. Our results showed high transcription levels of *dmrt1* and *psmc3ip* in the testis of *B. tsinlingensis* Li (Figure 7L,Q), suggesting their crucial roles as regulators of testicular development and spermatogenesis. 

In this study, significantly different KEGG enrichment results for DEGs were acquired from a comparative analysis of the testis and ovary of *B. tsinlingensis* Li, indicating their different regulatory mechanisms for gonadal development and gametogenesis. The results showed that mDEGs were most enriched in base excision repair (BER) and the notch signaling pathway (Figure 6A). BER is the main pathway for repairing DNA base damage and lesions [28,29]. Our results showed that BER was the most enriched pathway in the testis, consistent with the detection of high levels of BER-related gene-transcription products in male gonads [30,31,32,33]. Additionally, BER was an important pathway for maintaining genomic integrity in male germ cells [34]. Notch signaling components can always be detected in the Sertoli and germ cells of male gonads, and research has shown that the notch signaling pathway is crucial for regulating testicular early development, spermatogonial differentiation, and spermatogenesis [35,36,37]. Our results emphasized the importance of BER and notch signaling in the testicular reproductive regulation of *B. tsinlingensis* Li. 

The fDEGs were significantly enriched in the neuroactive ligand-receptor interaction pathway, the VEGF signaling pathway, and the estrogen signaling pathway. The neuroactive ligand-receptor interaction pathway can impact the synthesis of steroid hormones in the gonads via the HPG axis and plays an important regulatory role in ovulation [38,39]. Moreover, similar significant enrichment results were found in gonadal transcriptomic studies of chicken, sheep and goose gonads [40,41,42]. VEGF has been recognized as one of the most relevant angiogenetic factors in orchestrating folliculogenesis, and VEGF inhibition prevents follicle ovulation [43,44,45]. Moreover, estrogen has a significant regulatory role in oogenesis and ovulation [46]. Thus, our results revealed that these pathways may play vital roles in ovary development and gametogenesis in *B. tsinlingensis* Li. Additionally, many other biological pathways are also significantly enriched (Figure 6), and their functions require further investigation.

## 5. Conclusions

In this study, the first comparative transcriptome analysis between the testis and ovary of *B. tsinlingensis* Li was reported. A total of 22,864 DEGs were identified, including 17,231 male-biased DEGs and 5633 female-biased DEGs. Numerous genes related to reproductive development were explored, including *ccnb1*, *zp3*, *bmp15*, *dmrt1*, and *psmc3ip*. Biological pathways potentially involved in reproductive regulation were also identified. These pathways included base excision repair, the notch signaling pathway, neuroactive ligand-receptor interaction, the VEGF signaling pathway, and the estrogen signaling pathway. The results provide new insights into the reproductive development of *B. tsinlingensis* Li and will be beneficial for the further artificial breeding of this species.

## Figures and Tables

**Table 1:** Summary statistics for the gonadal transcriptome.

Sample	Raw Bases	Clean Bases	Raw Reads	Clean Reads	Mapped Reads（Ratio)	Error Rate (%)	Q30(%)	GC Content (%)
F_1	13,429,748,532	12,982,936,212	88,938,732	87,804,560	69,352,106 (78.98%)	0.025	94.2	49.59
F_2	11,051,384,074	10,653,775,488	73,187,974	72,275,082	57,395,498 (79.41%)	0.025	94.26	49.87
F_3	14,737,855,492	14,309,137,436	97,601,692	96,375,314	76,066,826 (78.93%)	0.0251	94.18	49.47
F_4	14,257,279,268	13,826,078,968	94,419,068	93,274,920	73,624,950 (78.93%)	0.0249	94.3	49.54
F_5	11,127,009,404	10,761,812,681	73,688,804	72,655,432	57,117,684 (78.61%)	0.0251	94.13	49.58
M_1	12,286,990,498	11,868,538,929	81,370,798	80,223,798	63,205,096 (78.79%)	0.0247	94.44	49.32
M_2	12,421,889,066	11,962,639,831	82,264,166	81,048,350	62,965,986 (77.69%)	0.025	94.17	50.2
M_3	11,909,431,910	11,467,120,995	78,870,410	77,846,102	61,366,132 (78.83%)	0.0245	94.69	50.05
M_4	12,657,506,144	12,189,596,284	83,824,544	82,737,530	64,825,426 (78.35%)	0.0245	94.68	49.86
M_5	12,159,281,644	11,741,956,029	80,525,044	79,461,734	61,365,664 (77.23%)	0.0247	94.53	50.09

**Table 2:** Evaluation of optimized results.

Type	Unigene	Transcript
200~500 (bp)	95,750 (57%)	123,592 (51%)
501~1000 (bp)	38,003 (23%)	53,103 (22%)
1001~2000 (bp)	18,088 (11%)	31,700 (13%)
2001~3000 (bp)	7510 (5%)	15,145 (7%)
3001~4000 (bp)	4002 (2%)	8236 (3%)
>4001 (bp)	4551 (3%)	9338 (4%)
Total number	167,904	241,114
Total base	140,382,062	240,137,939
Largest length (bp)	26,461	26,461
Smallest length (bp)	201	201
Average length (bp)	836.09	995.95
N50 length (bp)	1452	1943
E90N50 length (bp)	3227	3100
Fragment mapped percent (%)	60.856	77.793
GC percent (%)	45.79	46.68
TransRate score	0.27696	0.35206
BUSCO score	C:93.6% [S:78.8%; D:14.8%]	C:93.6% [S:78.8%; D:14.8%]

## Data Availability

The data sets supporting the results of this article have been submitted to GenBank and accession numbers are shown in the article.

## Supplementary Materials

The following supporting information can be downloaded at: https://www.mdpi.com/article/10.3390/ani13233690/s1, Figure S1: correlation analysis (A) and principal component analysis (B) for samples; Figure S2: GO classification (A) and KEGG pathway analysis (B) of DEGs; Table S1: list of primers used for quantitative RT-PCR validation; Table S2: species distribution of NR protein database annotations.
