# Supplementary material for: Comparative Transcriptome Analysis Reveals Sexually Dimorphic Gene Expression in the Gonads of Brachymystax tsinlingensis Li
Source: Animals (Basel). 2023 Nov 29;13(23):3690. doi: 10.3390/ani13233690 (PMC10705525; doi:10.3390/ani13233690)
Supplement: Supplementary file 1 [file animals-13-03690-s001.zip › table S1.pdf]

Table S1 List of primers used for quantitative RT-PCR validation

Gene	Primer sequences (5′–3′)	Amplicon length(bp)	Amplification efficiency
β-actin	F: ATCCTGACGGAGCGCGGTTACAG	112	100%
	R: TGCCCATCTCCTGCTCAAAGTCCA		
cyp19a	F: GACTTCGGAGAGTGCATACCA	127	92%
	R: TGCCAATACAGCTCAGACCC		
zp1	F: CAAGTCATCCAGAGGCCAGT	195	94%
	R: ATCCTTGGCCACCACAACAA		
trim25	F: CCCGCTCTGCAAGTATCAGT	198	95%
	R: TGCCTTGAGCCAAACCCATT		
parp7s	F: TCAACTGGATTCACGCAGCA	118	91%
	R: ATGGAGTTCGGGCAGGTTTT		
ccnb1	F: TGTGCAGTTCCAAAGGTCCAG	191	86%
	R: TCAGTAGCGCTCTGCTTACG		
zar1	F: CCGATCAGGATGGATCGAGAG	127	91%
	R: CCTTGGACGCACCAAACG		
zp3	F: AATGCGGGTAGTCCTTTGGG	168	91%
	R: CTTTGCTGCAACAGGCTCAG		
bmp15	F: ACAGTACGTCTGCTACGACC	138	92%
	R: GTGGACAAACGAGGCTCTCA		
nanos2	F: CGCCCCGACCCATTATTTC	115	103%
	R: CTCCTTCAGCTGGGTACAGG		
fshr	F: CCAGTGCTGAAACCCGCAT	182	92%
	R: GAAGGGGTTGGCACATGAGT		
dmrt1	F: TGAAGATGGTCGTTACCGGG	176	95%
	R: AGCGTGAGTAGAGCTTGAAGG		
tcte1	F: TGTGTGACGTCTCCTCATACG	101	105%
	R: GGGGTCAGTGGTATCAGGAATAA		
theg	F: AGCAACAGGGAGGTAAGTGC	110	97%
	R: ACGTAGAGCAGCTGTAAACCTT		
strbp	F: ACAAGGTACCACGGATGAACA	195	95%
	R: CAGGCTGGCAGTCTATTGCT		
cep4	F: AACAAACCGCCACTCAGACT	132	95%
	R: TCATGCTCAGACGCTCACTG		
pamc3ip	F: CCATCGACAGTCGCATCTCT	113	105%
	R: GGGTAGGGTGCTGATAGAGC		
pvrl3	F: AACATTACGCTGGGTTGCAG	180	91%
	R: CATGAAGGACAAGGGGTTCA		
sycp1	F: GGGCAGTTATCTGCTGCAATG	124	93%
	R: GCAGCTGATTAAAACTTGCTGA		
znt9	F: AAGTCCAACAAGAGTTCTGGCA	105	95%
	R: GGAGAGGGCCGTAGATTTCG		
